# Prevalence and characterization of *Escherichia coli* isolated from the Upper Oconee Watershed in Northeast Georgia

**DOI:** 10.1371/journal.pone.0197005

**Published:** 2018-05-08

**Authors:** Sohyun Cho, Lari M. Hiott, John B. Barrett, Elizabeth A. McMillan, Sandra L. House, Shaheen B. Humayoun, Eric S. Adams, Charlene R. Jackson, Jonathan G. Frye

**Affiliations:** 1 Department of Microbiology, University of Georgia, Athens, Georgia, United States of America; 2 Bacterial Epidemiology and Antimicrobial Resistance Research Unit, United States Department of Agriculture, Agricultural Research Service, Athens, Georgia, United States of America; USDA-ARS Salinity Laboratory, UNITED STATES

## Abstract

Surface waters are important sources of water for drinking, industrial, agricultural, and recreational uses; hence, contamination of water by fecal, pathogenic, or antimicrobial resistant (AR) bacteria is a major environmental and public health concern. However, very little data is available on prevalence of these bacteria in surface water throughout a watershed. This study aimed to characterize *Escherichia coli* present in the Upper Oconee Watershed, a mixed-use watershed in Athens, GA, USA for potential pathogenicity and AR. *E*. *coli* were enumerated by colony counts, cultured by enrichment and direct plating, and characterized by phylo-groups, diarrheagenic pathotypes, and antimicrobial susceptibility. From the analysis, 99.3% (455/458) of the total samples were positive for *E*. *coli* resulting in 496 isolates. *E*. *coli* counts were as high as 1.2×10^4^ CFU/100 ml, which is above the United States Environmental Protection Agency (U.S. EPA) threshold for recreational water (235 CFU/100 ml based on a one-time measurement). Phylo-groups B2 (31.7%; 157/496) and B1 (30.8%; 153/496) were the most prevalent among the isolates. Enteropathogenic *E*. *coli* (EPEC) (19/496) and Shiga toxin-producing *E*. *coli* (STEC) (1/496) were the only diarrheagenic pathotypes detected. AR was observed in 6.9% (34/496) of the isolates, 15 of which were multidrug resistant (MDR; resistance to two or more classes of antimicrobials). Tetracycline resistance was most often detected (76.5%; 26/34), followed by ampicillin (32.4%; 11/34), streptomycin (23.5%; 8/34), sulfisoxazole (23.5%; 8/34), and nalidixic acid (14.7%; 5/34). Results from this study showed that *E*. *coli* is prevalent in high levels in the Upper Oconee Watershed, suggesting possible widespread fecal contamination. The presence of pathogenic, AR *E*. *coli* in the watershed indicates that environmental water can serve as a reservoir of resistant bacteria that may be transferred to humans through drinking and recreational activities.

## Introduction

*Escherichia coli*, which normally resides in the intestinal flora of warm-blooded animals, including humans, is ubiquitous in the environment and has been used as an indicator of fecal contamination to assess the safety and quality of water [1]. Although most *E*. *coli* strains are harmless, certain strains are pathogenic and cause diseases such as watery diarrhea, bloody diarrhea, urinary tract infection, meningitis, and sepsis, which can lead to death [2, 3]. The normally zoonotic bacterial pathogen has been responsible for waterborne outbreaks in humans through contaminated drinking and recreational water not only in developing countries, but also in industrialized countries [4–9]. Environmental water sources are prone to bacterial pollution from both humans and animals. Possible human sources include discharge of wastewater, sewage leaks, and failing septic tanks, as well as municipal, residential, medical, and industrial waste facilities. Animal sources include runoffs from animal farms, land application of animal manure, pet wastes from parks, and wildlife such as raccoons and deer. Since surface waters are often used for recreational and drinking purposes, the presence of pathogenic *E*. *coli* in waterways may increase the likelihood of human infections after exposure to these water sources.

The Upper Oconee Watershed, located in the Southern Piedmont of Georgia, USA, is a historically agricultural region that has experienced rapid urban development. While two-thirds of the watershed still remains undeveloped with rural residential, forest and agricultural lands, the remaining land areas have transitioned to urban and suburban residential areas [10]. The Upper Oconee Watershed is not only impacted by dense residential development, industrialization, and sporadic sewer spills, but also includes land areas heavily devoted to agriculture, including poultry, dairy cattle, and beef cattle production [11, 12]. Since the watershed provides water for municipal and recreational purposes, monitoring the water quality is a public health concern. The Upper Oconee Watershed Network (UOWN) is a nonprofit organization dedicated to protecting streams and rivers within the Upper Oconee Watershed [13]. Since January 2000, the UOWN has been monitoring the surface water and its reporting indicates recurrent fecal contamination of the surface water within the watershed as evidenced by high fecal coliform and *E*. *coli* levels [11, 14–16].

The goal of this study was to investigate seasonal and spatial prevalence and characteristics of *E*. *coli* present in the Upper Oconee Watershed in and around Athens, Georgia. Since previous reports only included data on fecal indicator levels within the surface water of the Upper Oconee Watershed [11, 14–16], the present study attempted to further examine surface water quality for recreational and drinking purposes by investigating each *E*. *coli* isolate for its potential to cause disease and its antimicrobial resistance (AR). Environmental water samples were collected each season for two years and fecal contamination was determined by enumerating *E*. *coli* colony counts. *E*. *coli* was isolated from each water sample and characterized for phylo-group, pathotype, and AR phenotype. Susceptibility testing with 14 antimicrobials that are largely used for treating human and animal infections was used to determine AR phenotypes, because AR *E*. *coli* could be a public health concern as they can potentially restrict treatment options in the event of an infection. This study provides unique data on *E*. *coli* prevalence and characteristics in a mixed-use watershed that is representative of what residents of rural, urban, and suburban areas may be exposed to through the recreational, agricultural, and municipal use of surface water. Although there have been several studies on *E*. *coli* in surface water, most of these studies focused on a single waterway rather than a watershed, and the few studies of watersheds were usually limited in sampling events, sites, or time period. This study is unique in that it provides data on an entire watershed sampled over two years and numerous sampling sites.

## Materials and methods

### Sampling area

**Fig 1:**
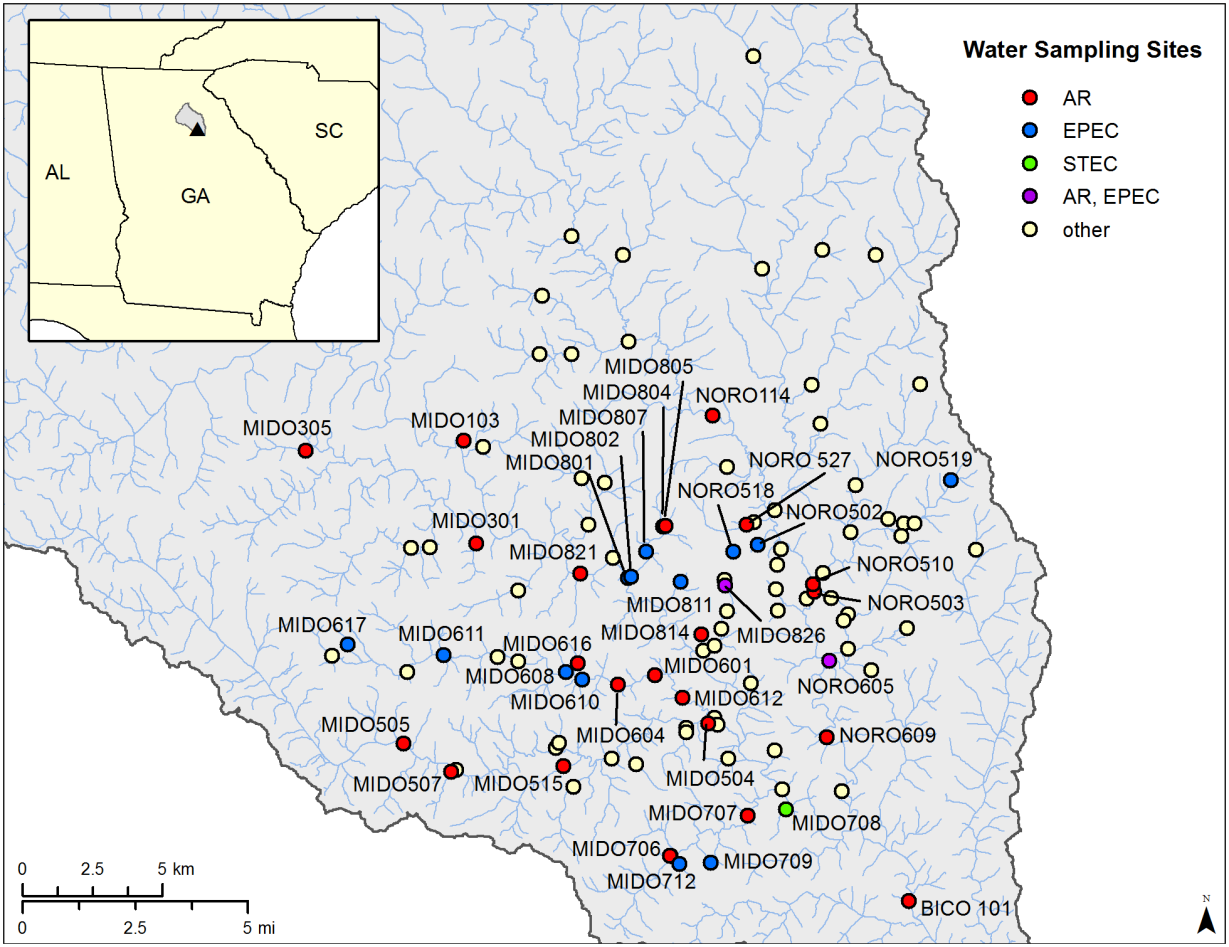
Map of water sampling sites in the Upper Oconee Watershed. The map of the Upper Oconee Watershed in Georgia with the enlarged map of the study area. Sampling sites where AR *E*. *coli* were isolated are in red, where EPEC were isolated are in blue, where STEC was isolated is in green, and where both AR *E*. *coli* and EPEC were isolated are in purple (sites labeled). Other sites, where all *E*. *coli* isolates were pan-susceptible and non-pathogenic, are in yellow.

The rivers and streams sampled in this study were located in the Upper Oconee Watershed (USGS Cataloging unit 03070101). As previously described [17], the study area is approximately 600 km^2^ and located within the lower Appalachian Piedmont of Northeastern Georgia, USA. Sampling sites were located along the North Oconee River (NORO), Middle Oconee River (MIDO), and their tributaries. A part of the study area is developed and densely populated consisting of urban residential areas that depend primarily on community sewers for effluent wastewater [17]. Other parts of the area mainly consist of forested, agricultural, and rural or suburban residential areas with poultry, dairy, and beef farming, largely depending on private septic systems for effluents [17]. Sampling sites were selected by the UOWN to represent a range of land uses (Fig 1). Maps and site descriptions for the sampling sites are available from the UOWN website [13] and in Fig 1. The exact locations of the sites with the GPS coordinates are in S1 Table. No specific permissions were required to collect water samples from these public access sites and no wildlife, endangered, or protected species were involved in this study.

### Water collection and enumeration of *E*. *coli* in water samples

One-liter water samples were collected once each season for two years from 2015 Winter to 2016 Fall at different locations in the Upper Oconee Watershed with the assistance of the UOWN volunteers. The number of water samples collected each time varied from 30 to 100, depending on available manpower and access to the sampling sites. Samples were stored at 4°C until processing the next day.

*E*. *coli* counts were enumerated in duplicates using Petrifilm^™^ *E*. *coli*/Coliform count plates (3M^™^, St. Paul, MN, USA) according to manufacturer’s directions. *E*. *coli* enumeration was carried out before the filtration of water samples by inoculating two Petrifilm^™^ plates with 1 ml of water each per sample. Plates were incubated at 37°C, and colonies were enumerated after 18–20 h of incubation. *E*. *coli* counts were averaged from the duplicate plates and expressed as CFU/100 ml.

### Isolation and identification of *E*. *coli*

Filtration of water samples was performed as previously described [17]. Briefly, 0.5 g of cellulose filter powder (Aqua Dew^™^, Lahore, Pakistan) was added to water samples, and the water samples were filtered onto 47-mm glass fiber filters of 0.3 μm pore size (Pall Corporation, Ann Arbor, MI, USA), which had been preloaded with another 0.5 g of cellulose filter powder suspended in 15 ml of sterile water. The filter, along with the filter powder, was incubated in 25 ml of 1X buffered peptone water (BD Difco^™^, Franklin Lakes, NJ, USA) for non-selective pre-enrichment of samples. All overnight samples were incubated at 37°C for 18–20 h.

For *E*. *coli* isolation, 0.1 ml of each peptone broth enrichment was streaked on a CHROMagar ECC agar plate (CHROMagar Microbiology, Paris, France). One year of samples were also plated onto CHROMagar O157 agar plates (CHROMagar Microbiology). However, this medium yielded no O157 isolates and its use was discontinued (data not shown). In addition to CHROMagar ECC agar plates, m-TEC agar plates (HiCrome^™^, Mumbai, India), which are normally used in the EPA method, were also used for isolation of *E*. *coli* during the 2015 Winter and Summer seasons. However, the m-TEC agar did not give consistent results and often failed to yield *E*. *coli* isolates, therefore that method was also discontinued. After a 37°C overnight incubation, one colony having the typical appearance of *E*. *coli* was selected from each positive plate. Presumptive positive *E*. *coli* isolates were then confirmed using the VITEK^®^2 System and the VITEK 2 GN colorimetric identification cards (BioMérieux, Durham, NC, USA) according to manufacturer’s directions. All bacterial isolates were stored in LB (Luria-Bertani) broth (BD Difco^™^), containing 30% glycerol at -80°C.

### Phylogenetic analysis and identification of diarrheagenic pathotypes of *E*. *coli*

For genotypic profiling of *E*. *coli*, phylo-group and diarrheagenic pathotype identifications were performed. *E*. *coli* were grouped into eight phylo-groups, A, B1, B2, C, D, E, F, and cryptic clade I, using the quadruplex phylo-typing PCR method as previously described [18]. *E*. *coli* ATCC 25922 and ATCC BAA-196 were used as control strains. The diarrheagenic pathotypes were determined using PCR methods for detection of the following genes: pCVD, *ipaH*, *est*, *elt*, *stx1*, *stx2*, *eaeA*, and *hlyA*. Using previously described methods, *E*. *coli* isolates were characterized as enteroaggregative *E*. *coli* (EAEC) (pCVD+) [19], enteroinvasive *E*. *coli* (EIEC) (*ipaH*+) [20], enterotoxigenic *E*. *coli* (ETEC) (*est*+, *elt*+) [20], enterohemorrhagic *E*. *coli* (EHEC) (*stx1*+ and/or *stx2*+, *eaeA*+, *hlyA*+) [19], enteropathogenic *E*. *coli* (EPEC) (*eaeA*+, *stx1*-, *stx2*-, *hlyA*-) [21], and Shiga toxin-producing *E*. *coli* (STEC) (*stx1*+ and/or *stx2*+, *eaeA*-, *hlyA*-) [21]. The following *E*. *coli* strains were included as positive control strains: ATCC 29552 (EAEC), ATCC 35401 (ETEC), ATCC 43893 (EIEC), and ATCC 43895 (EPEC, EHEC, and STEC). The STEC isolate was tested for serogroups O111 and O157 using a method previously described [21]. PCR was performed as described in the given references using whole-cell templates that were prepared by suspending a single bacterial colony in 200 μl of sterile deionized water. Amplified PCR products were then analyzed by electrophoresis on 2% agarose gel and visualized by staining with ethidium bromide.

### Antimicrobial susceptibility testing

Minimum inhibitory concentrations (MIC) of all *E*. *coli* isolates were determined by broth-microdilution using the Sensititre^™^ semi-automated antimicrobial susceptibility system (TREK Diagnostic Systems Inc., Cleveland, OH, USA) and the Sensititre^™^ custom National Antimicrobial Resistance Monitoring System (NARMS) plate CMV3AGNF according to manufacturer’s directions. MICs of the isolates for the 14 antimicrobials were determined, and each isolate was classified as resistant, intermediate, or susceptible to the antimicrobials tested using the breakpoints set by Clinical and Laboratory Standards Institute (CLSI) [22]. For azithromycin, without CLSI approved breakpoints, the epidemiological cutoff value for wild-type *Salmonella* (MIC > 16 μg/ml) was used [23, 24]. The 14 antimicrobials and the breakpoints (μg/ml) for determining resistances were as follows: amoxicillin/clavulanic acid (≥ 32/16), ampicillin (≥ 32), azithromycin (> 16), cefoxitin (≥ 32), ceftiofur (≥ 8), ceftriaxone (≥ 4), chloramphenicol (≥ 32), ciprofloxacin (≥ 1), gentamicin (≥ 16), nalidixic acid (≥ 32), streptomycin (≥ 64), sulfisoxazole (≥ 512), tetracycline (≥ 16), and trimethoprim/sulfamethoxazole (≥ 4/76). For the analysis, isolates identified as intermediate were considered susceptible to the drug. *E*. *coli* ATCC 25922, *Pseudomonas aeruginosa* ATCC 27853, *Enterococcus faecalis* ATCC 29212, and *Staphylococcus aureus* ATCC 29213 were used as control strains for MIC determination.

## Results

### Prevalence of *E*. *coli*

A total of 458 water samples were collected from eight seasonal sampling events. The sampling site locations are shown on the map in Fig 1 and listed in S1 Table with each site’s GPS coordinates. The number of sampling sites positive for *E*. *coli* and the number of isolates recovered from the sites are shown in Table 1. *E*. *coli* was recovered from 99.3% (455/458) of the total sampling sites, with the recovery rate for each sampling ranging from 96.7% to 100.0%, and a total of 496 *E*. *coli* were isolated. Although only one colony was selected from each positive plate, higher number of isolates than the number of sites is due to the use of mTEC agar in addition to CHROMagar ECC agar. Multiple media were used for the isolation of *E*. *coli* in order to test the efficacy of each media.

**Table 1:** *E*. *coli* isolates recovered from sampling sites.

Sampling season (no. of samples)	% of positive sites (no. of isolates recovered)
Winter 2015 (30)	96.7 (56)^a^
Spring 2015 (100)	99.0 (99)
Summer 2015 (33)	97.0 (46)^a^
Fall 2015 (59)	100.0 (59)
Winter 2016 (41)	100.0 (41)
Spring 2016 (87)	100.0 (87)
Summer 2016 (27)	100.0 (27)
Fall 2016 (81)	100.0 (81)

^a^ Higher numbers of isolates than the numbers of sites are due to the use of several media to recover *E*. *coli*

**Fig 2:**
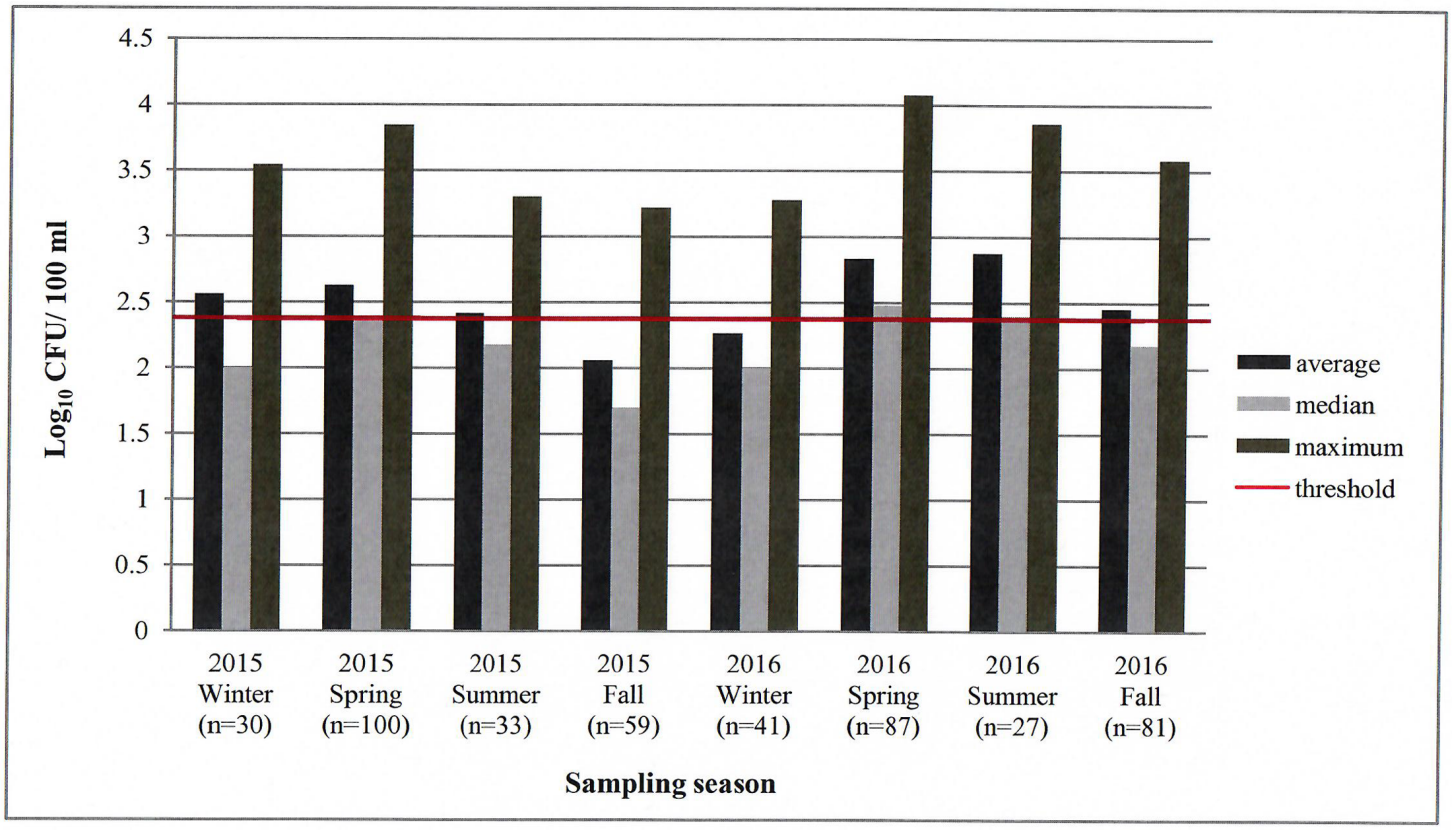
Seasonal distribution of *E*. *coli* in the Upper Oconee Watershed in colony forming units [CFU] per 100 ml. X-axis represents each sampling season with the numbers in parenthesis indicating the total number of water samples. Y-axis represents the *E*. *coli* counts in log_10_ CFU/100 ml. The threshold represents the EPA threshold for water quality for recreational purposes.

*E*. *coli* colony count results for each season is shown in Fig 2 in log_10_ CFU/100 ml. Approximately 39% (177/458) of the total samples exceeded the United States Environmental Protection Agency (U.S. EPA) threshold for recreational activities, which is 235 CFU/100 ml based on a one-time measurement [1]. The average of the *E*. *coli* counts per season was above the threshold during six out of the eight sampling seasons, while the median *E*. *coli* counts exceeded the threshold only in Spring and Summer seasons of 2016. The *E*. *coli* counts were as low as undetectable (detection limit of 50 CFU/100 ml) and as high as 1.2×10^4^ CFU/100 ml. The number of sampling sites that exceeded 235 CFU/100 ml for each sampling event is shown in Table 2.

**Table 2:** Sites with *E*. *coli* counts exceeding the U.S. EPA threshold for each sampling event^a^.

Sampling season (total no. of sites sampled)	Winter 2015	Spring 2015	Summer 2015	Fall 2015	Winter 2016	Spring 2016	Summer 2016	Fall 2016
	(30)	(100)	(33)	(59)	(41)	(87)	(27)	(81)
no. of sites that exceeded the EPA threshold	10	50	8	4	9	51	18	27

^a^ U.S. EPA threshold for recreational activities = 235 CFU/ 100 ml based on a one-time measurement

### Identification and characterization of *E*. *coli*

Phylo-groups and diarrheagenic pathotypes of *E*. *coli* isolates recovered from surface water are shown in Tables 3 and 4. Using the quadruplex phylo-typing method, six phylo-groups (A, B1, B2, C, E, and F) were identified while three isolates could not be assigned a phylo-group (unknown; U). The most prevalent groups were B2 (31.7%; 157/496) and B1 (30.8%; 153/496). Fewer isolates were identified as groups E (23.2%; 115/496), A (6.7%; 33/496), F (4.6%; 23/496), and C (2.4%; 12/496).

**Table 3:** Phylo-groups and diarrheagenic pathotypes of *E*. *coli* isolated from surface water.

	Season (total number of isolates)	number of isolates							
		Winter	Spring	Summer	Fall	Winter	Spring	Summer	Fall
		2015	2015	2015	2015	2016	2016	2016	2016
		(56)	(99)	(46)	(59)	(41)	(87)	(27)	(81)
Phylo-group	A	6	7	2	4	2	7	1	4
	B1	16	21	21	21	19	21	9	25
	B2	16	40	5	20	11	39	8	18
	C	4	3	1	0	1	2	0	1
	E	12	25	15	11	6	14	6	26
	F	2	3	2	3	1	4	2	6
	U	0	0	0	0	1	0	1	1

**Table 4:** Antimicrobial resistant and pathogenic *E*. *coli* isolated from surface water, phylo-group, and sample site location from Fig 1.

Season	Isolate ID	AR pattern^a^	Phylo-group	Location
Winter 2015	3 mTEC	AmpCipNalStrSulTetTri	B1	MIDO 103
	13 mTEC	Tet	F	MIDO 612
	15 mTEC	Tet	B1	MIDO 616
	25 mTEC	Tet	B1	NORO 510
	27 mTEC	StrSulTet	C	NORO 605
	13 ECC	Tet	C	MIDO 612
	25 ECC	Tet	A	NORO 510
	27 ECC	Tet	B1	NORO 605
	29 ECC	Tet	C	NORO 609
Spring 2015	40 ECC	StrSulTet	B1	MIDO 504
	65 ECC	Tet	C	MIDO 706
	66 ECC	Nal	C	MIDO 707
	95 ECC	Tet	E	NORO 114
	107 ECC	Amp	E	NORO 503
Summer 2015	159c mTEC	Tet	E	MIDO 826
	161 ECC	Amp	B2	NORO 503
Fall 2015	164 ECC	AmpNal	B2	BICO 101
	171 ECC	Tet	B1	MIDO 505
	192 ECC	ChlStrSulTet	B2	MIDO 707
	205 ECC	Tet	B2	MIDO 814
	207 ECC	Amp	E	MIDO 821
Winter 2016	238 ECC	AmpAziStrSulTetTri	E	MIDO 604
	255 ECC	Tet	B2	NORO 503
Spring 2016	264 ECC	SulTet	A	BICO 101
	274 ECC	AmpStrTet	A	MIDO 507
	279 ECC	StrSulTet	B1	MIDO 515
	280 ECC	AmpNal	B2	MIDO 601
	281 ECC	Tet	B1	MIDO 604
	303 ECC	AmoAmpFoxTioAxoGen	B2	MIDO 805
	339 ECC	AmoTet	B1	NORO 527
Summer 2016	353 ECC	StrTet	B1	MIDO 305
	367 ECC	AmpTioAxoNal	B2	MIDO 826
Fall 2016	381 ECC	AziSulTetTri	C	MIDO 103
	382 ECC	Tet	F	MIDO 301
Season	Isolate ID	Pathotype^b^	Phylo-group	Location
Winter 2015	19 mTEC	EPEC	B2	MIDO 801
	12 ECC	EPEC	B1	MIDO 611
Spring 2015	74 ECC	EPEC	B2	MIDO 804
	119 ECC	EPEC	B2	NORO 519
	124 ECC	EPEC	B2	NORO 605
Summer 2015	152 ECC	EPEC	B1	MIDO 801
	154 ECC	EPEC	C	MIDO 804
	159 ECC	EPEC	A	MIDO 826
Fall 2015	193 ECC	STEC	B1	MIDO 708
	194 ECC	EPEC	B2	MIDO 709
	195 ECC	EPEC	B2	MIDO 712
	201 ECC	EPEC	B2	MIDO 807
	202 ECC	EPEC	B2	MIDO 811
	219 ECC	EPEC	B2	NORO 605
Winter 2016	251 ECC	EPEC	B2	NORO 502
	253 ECC	EPEC	B2	NORO 518
Summer 2016	358 ECC	EPEC	B2	MIDO 610
Fall 2016	394 ECC	EPEC	B2	MIDO 608
	401 ECC	EPEC	B2	MIDO 617
	411 ECC	EPEC	B2	MIDO 802

^a^ amoxicillin/clavulanic acid (Amo), ampicillin (Amp), azithromycin (Azi), cefoxitin (Fox), ceftiofur (Tio), ceftriaxone (Axo), chloramphenicol (Chl), ciprofloxacin (Cip), gentamicin (Gen), nalidixic acid (Nal), streptomycin (Str), sulfisoxazole (Sul), tetracycline (Tet), trimethoprim/sulfamethoxazole (Tri)

^b^ EPEC: enteropathogenic *E*. *coli* and STEC: Shiga toxin-producing *E*. *coli*

Out of a total of 496 *E*. *coli* isolates, 19 EPEC and 1 STEC, positive for *stx2*, were detected (Table 4). The STEC isolate did not belong to serogroup O157 or O111. The majority of EPEC (15/19) isolates belonged to phylo-group B2 while the STEC belonged to phylo-group B1. No EAEC, EIEC, EHEC, or ETEC were detected. Locations where EPEC and the STEC were isolated are indicated on Fig 1.

Most of the *E*. *coli* isolates were susceptible to the 14 drugs tested with only 6.9% (34/496) of the isolates exhibiting resistance to any of the drugs. These 34 AR *E*. *coli* were isolated from 24 sampling sites; eight of the sites had two AR *E*. *coli* isolated from them, and one site had three AR *E*. *coli* isolated from it. For this study, we considered resistance to two or more classes of antimicrobials as multidrug resistance (MDR). We chose this cut off to indicate resistance to multiple classes of antimicrobials rather than resistance to multiple antimicrobials, which if in the same class could be conferred by a single gene or genetic mutation. Therefore, using this definition helps to indicate that an isolate which is resistant to multiple classes of antimicrobials may have multiple mechanisms of AR. MDR was observed in 15 of the isolates. Eleven different MDR patterns were detected, including one isolate resistant to seven antimicrobials (Table 4). Resistance to all of the 14 drugs tested was observed in the *E*. *coli* isolates from this study. Resistance to tetracycline was the most prevalent (76.5%; 26/34), followed by resistance to ampicillin (32.4%; 11/34), streptomycin (23.5%; 8/34), sulfisoxazole (23.5%; 8/34), and nalidixic acid (14.7%; 5/34). Locations from which AR *E*. *coli* were isolated are shown on Fig 1. Interestingly, none of the EPEC or STEC isolates was resistant to any of the antimicrobials tested.

## Discussion

### Prevalence of *E*. *coli* in the watershed

The results of this study indicated that *E*. *coli* was highly prevalent in the Upper Oconee Watershed as *E*. *coli* was isolated from almost every water site sampled each season. Due to the ubiquity of *E*. *coli*, no seasonal variations in presence was detectable; however, colony counts did vary. *E*. *coli* colony counts were determined using the 3M^™^ Petrifilm^™^ method, which is often used for volunteer-based water quality monitoring for its effectiveness, cost efficiency, and simplicity of use and storage [25–27]. Consistent with the previous reports on the water quality of the Upper Oconee Watershed [11, 14–16], high *E*. *coli* counts were detected in the present study, which is evidence for widespread fecal contamination within the watershed. The *E*. *coli* counts of the water samples often exceeded the EPA threshold for recreational activities such as swimming and water skiing, which is 235 CFU/100 ml based on a one-time measurement [1]. The *E*. *coli* counts exceeded the threshold more often in the spring and summer seasons than in the fall and winter seasons, likely due to warmer water temperatures supporting growth of this enteric bacterium. In general, rural streams had acceptable *E*. *coli* counts while urban and suburban streams had higher levels of *E*. *coli* counts, which may be attributed to surface runoff from built infrastructure, leaking sewer lines, and failing septic systems. *E*. *coli* counts exceeding 10^3^ CFU/100 ml were frequently observed which warrant special attention as it may indicate direct sewage contamination [25].

### Pathogenic potential of *E*. *coli* in the watershed

Phylo-grouping PCR results showed that a third of all the *E*. *coli* isolates belonged to phylo-group B2, which is known to be associated with virulence and accounts for the majority of extra-intestinal infections [28]. The second most prevalent group was B1, to which commensal *E*. *coli* typically belong [28]. None of the isolates belonged to phylo-group D, which was contrary to previous reports that have shown a sizeable percentage of the group D isolates in environmental water samples [29–31]. The percentages of *E*. *coli* isolates that belonged to phylo-group D were 25.0% in the Mid-Atlantic region of the U.S. [29], 10.8% in the Yeongsan River basin of South Korea [30], and as high as 80.0% in the St. Clair River and Detroit River [31]. However, as opposed to the previous studies that used a triplex PCR developed by Clermont *et al*. in 2000 [32], the current study used a quadruplex PCR which was developed by Clermont *et al*. in 2012 as an improvement of the previous PCR method [18]. With the refined knowledge of *E*. *coli* phylogenetic group structure using multi-locus sequence type (MLST) data, new phylo-groups C, E, F, and *Escherichia* clade I were recognized and included in the phylo-typing PCR method, demonstrating the significant percentage of incorrect phylo-group assignment of *E*. *coli* strains using the previous triplex PCR [18, 33]. Unfortunately, few studies in the literature have yet to use the quadruplex PCR method to characterize *E*. *coli* isolates from surface water. Therefore, it is unclear if it is the use of the different methods that has resulted in the difference in the percentages of the phylo-groups or if the phylo-groups follow a region- or site- specific pattern.

EPEC was rarely isolated in water samples in this study, with a total of 19 EPEC isolates detected. Humans are the main reservoir of EPEC, which causes watery diarrhea primarily in children under two years old [2]. Although this strain of *E*. *coli* persists in developing countries as a cause of diseases [2], EPEC is no longer a public threat in developed countries and only 30 cases of EPEC infections were confirmed in the U.S. from 2014 to 2016 [2, 34–36]. A majority of EPEC belonged to phylo-group B2, which was consistent with previous studies that have reported that B2 strains tend to harbor more virulence determinants than the strains that belong to other phylo-groups [37–39]. Only one STEC was detected in any water sample during any season in the present study. Because this *stx2*-positive isolate does not have any other virulence factor, such as *eaeA*, and cattle are known to be a vast reservoir of STEC [3], it is very probable that this STEC isolate originated from an animal source. *E*. *coli* O157, responsible for most human infections among the STECs in developed countries [3, 21], has often been detected from surface water [40, 41]. *E*. *coli* O157 outbreaks involving surface water contaminated with human and animal feces have been previously documented as well [5, 7, 9]. However, the present study did not detect any *E*. *coli* O157 isolates. While EAEC, EHEC, EIEC, and ETEC have been previously identified in surface water [42], none were detected in the Upper Oconee Watershed similar to findings for the St. Clair and Detroit rivers [31]. Locations from which the EPEC and STEC isolates were collected are indicated in Fig 1. Overall, not many diarrheagenic strains of *E*. *coli* have been identified from the Upper Oconee Watershed; nevertheless, the isolation of EPEC and STEC from the surface water does suggest potential exposure of environmental water to fecal contamination of human and/or animal origin.

### Antimicrobial resistant *E*. *coli* in the watershed

Only a small portion of water samples harbored *E*. *coli* resistant to any of the 14 drugs tested. However, it is important to note that our isolation method did not use antimicrobials to select for resistant strains; therefore, the level of 6.9% resistant *E*. *coli* likely represented a true level of resistant *E*. *coli* in the watershed, which is not trivial considering the high level of some of the sample colony counts. Resistance was observed most often to tetracycline, followed by ampicillin, streptomycin, sulfisoxazole, and nalidixic acid. The high resistance rate to tetracycline has been previously reported in other studies [43–45], indicating that the resistance to tetracycline is prevalent in environmental water. This observation was expected as tetracycline is one of the most widely used antimicrobials for treatment of human and animal infections as well as the historic use for agricultural purposes as growth promoters [46, 47]. The frequency of resistance to the antimicrobials listed above corresponded with findings from other regions, while the prevalence of AR in *E*. *coli* from the Upper Oconee Watershed was less than expected based on levels seen in other environmental water sources [38, 43–45]. High levels of AR to a variety of antimicrobials have been reported for *E*. *coli* isolates from aquatic environment, as high as 82% in other regions of the country [38, 43–45], and as high as 100% in other parts of the world [48–50]. The difference in the level of AR with the *E*. *coli* isolates from this study may be partially due to the antimicrobial drugs chosen for testing. Variances in the therapeutic drugs used and the levels of fecal contamination may also have contributed to the difference.

AR *E*. *coli* were mostly recovered from residential areas. The exact locations of the sites with the GPS coordinates are in S1 Table and locations where AR positive samples were collected are indicated in Fig 1. The city of Athens, which encompasses Clarke County and some parts of Oconee and Jackson Counties, is served by a sewer system with surprisingly high cases of sewage problems [51]. These include the case of an unknown amount of improperly treated wastewater being discharged into a creek over an unknown period of time [52], increasing the likelihood of isolating AR *E*. *coli* of human source within the residential land areas. A few other sites from where AR *E*. *coli* were recovered were located near agricultural operations i.e. MIDO 103, MIDO 305, MIDO 505, and MIDO 507. There were cattle pastures, a poultry farm, and a small horse farm near the sampling sites which could have been potential sources of AR *E*. *coli* isolated.

McNutt Creek flows through suburban residential and commercial areas of Athens. Several sampling sites were located along the creek and its tributaries, and the quality of water has been shown to be a concern due to high *E*. *coli* counts and the presence of AR and pathogenic *E*. *coli*. Four out of 19 EPEC isolates and six out of 34 AR *E*. *coli* isolates were collected from McNutt Creek alone. McNutt Creek is on the EPA Total Maximum Daily Load (TMDL) 303(d) list of Impaired Waters in terms of fecal coliform due to nonpoint sources and urban runoff [53]. Continuous monitoring of the creek to track the sources of contamination is required to gather data for the improvement of the creek’s water quality.

Although there are several papers on *E*. *coli* from surface waters of different locations around the world, relatively little has been studied about *E*. *coli* prevalence, pathotypes, and antimicrobial susceptibility in surface waters of a mixed-use watershed in the U.S. One of the most similar studies was reported by Ibekwe *et al*. who collected samples from 20 sites quarterly over a twelve-month period from the middle Santa Anna River in Southern California, USA [45]. However, the watershed drained by that river is dominated by a large area of cattle farms and discharges from three wastewater treatment plants. In addition, that study also sampled sediments thus representing the environment in and around the river, whereas our study focused on bacteria within the moving water column indicative of what residents would be exposed to by recreational, agricultural, and municipal use of surface water. Our study area was diverse and represented land use from the undeveloped forest, agricultural and rural residential lands (about 62% of the land in the watershed) to densely developed industrial and suburban- residential lands (about 38%). Water samples were collected quarterly for two years from 100 different sampling sites that encompassed the entire watershed, incorporating not only relatively pristine streams but also streams with a history of human impacts, such as runoff from agricultural activities as well as contaminated effluents from wastewater treatment plants, discharges from failing septic systems, and sewer line leaks. As approximately half of the U.S. population lives in suburban areas [54], this mixed-use watershed may be a good representation of the conditions many U.S. residents are exposed to by surface water used for recreational, agricultural, and municipal purposes.

This study has shown the seasonal and spatial prevalence and characteristics of *E*. *coli* in surface water of the Upper Oconee Watershed, Athens, GA, including the presence of pathogenic and AR *E*. *coli*. *E*. *coli* resistant to therapeutic drugs were not highly prevalent in the environment and these commensal bacteria may not appear to be a risk to public health. However, *E*. *coli* is known to harbor AR genes on plasmids, transposons, and integrons, and these mobile genetic elements can be transferred between organisms of the same species or different genera through horizontal gene transfer [55, 56]. Therefore, further studies are required to assess risks associated with *E*. *coli* harboring AR genes and the potential of transferring these genes to other bacteria, including commensal *E*. *coli* and other bacterial pathogens which could have a detrimental impact on public health.

## Supporting information

S1 Table Master file of the *E*. *coli* isolated from the Upper Oconee Watershed. ^a^ amoxicillin/clavulanic acid (Amo), ampicillin (Amp), azithromycin (Azi), cefoxitin (Fox), ceftiofur (Tio), ceftriaxone (Axo), chloramphenicol (Chl), ciprofloxacin (Cip), gentamicin (Gen), nalidixic acid (Nal), streptomycin (Str), sulfisoxazole (Sul), tetracycline (Tet), trimethoprim/sulfamethoxazole (Tri). ^b^EPEC: enteropathogenic *E*. *coli* and STEC: Shiga toxin-producing *E*. *coli*.
(PDF)
