# Supplementary material for: Prevalence and characterization of Escherichia coli isolated from the Upper Oconee Watershed in Northeast Georgia
Source: PLoS One. 2018 May 8;13(5):e0197005. doi: 10.1371/journal.pone.0197005 (PMC5940194; doi:10.1371/journal.pone.0197005)
Supplement: S1 Table — a amoxicillin/clavulanic acid (Amo), ampicillin (Amp), azithromycin (Azi), cefoxitin (Fox), ceftiofur (Tio), ceftriaxone (Axo), chloramphenicol (Chl), ciprofloxacin (Cip), gentamicin (Gen), nalidixic acid (Nal), streptomycin (Str), sulfisoxazole (Sul), tetracycline (Tet), trimethoprim/sulfamethoxazole (Tri). bEPEC: enteropathogenic E. coli and STEC: Shiga toxin-producing E. coli. (PDF) [file pone.0197005.s001.pdf]

Season	Isolate ID	Location	GPS coordinates	media	E. coli counts	Phylogenetic group	Pathotype	AR pattern
2015 Winter	1 mTEC	BICO 201	33.969265, -83.303852	mTEC	350	E	N/A	Pan-susceptible
	2 mTEC	MIDO 101	33.9987, -83.4954	mTEC	0	B1	N/A	Pan-susceptible
	3 mTEC	MIDO 103	34.0006, -83.503	mTEC	150	B1	N/A	AmpCipNalStrSulTetTri
	4 mTEC	MIDO 305	33.996, -83.5639	mTEC	300	B2	N/A	Pan-susceptible
	5 mTEC	MIDO 601	33.926339, -83.426795	mTEC	0	B2	N/A	Pan-susceptible
	6 mTEC	MIDO 604	33.9232, -83.441	mTEC	550	E	N/A	Pan-susceptible
	7 mTEC	MIDO 605	33.931, -83.4877	mTEC	50	E	N/A	Pan-susceptible
	8 mTEC	MIDO 606	33.9298, -83.4798	mTEC	200	B1	N/A	Pan-susceptible
	10 mTEC	MIDO 609	33.9301, -83.5517	mTEC	450	B1	N/A	Pan-susceptible
	11 mTEC	MIDO 610	33.9245, -83.4549	mTEC	100	E	N/A	Pan-susceptible
	12 mTEC	MIDO 611	33.9313, -83.5086	mTEC	50	B1	N/A	Pan-susceptible
	13 mTEC	MIDO 612	33.9194, -83.4159	mTEC	0	F	N/A	Tet
	14 mTEC	MIDO 613	33.9255, -83.5226	mTEC	100	C	N/A	Pan-susceptible
	15 mTEC	MIDO 616	33.9296, -83.4567	mTEC	0	B1	N/A	Tet
	17 mTEC	MIDO 704	33.903, -83.3797	mTEC	100	B2	N/A	Pan-susceptible
	18 mTEC	MIDO 708	33.900855, -83.379822	mTEC	0	B2	N/A	Pan-susceptible
	19 mTEC	MIDO 801	33.957765, -83.438489	mTEC	200	B2	EPEC	Pan-susceptible
	20 mTEC	MIDO 802	33.958, -83.4368	mTEC	100	E	N/A	Pan-susceptible
	21 mTEC	MIDO 804	33.9743, -83.425	mTEC	200	B2	N/A	Pan-susceptible
	22 mTEC	MIDO 805	33.9746, -83.4241	mTEC	100	B2	N/A	Pan-susceptible
	23 mTEC	NORO 503	33.9545, -83.3659	mTEC	0	B1	N/A	Pan-susceptible
	24 mTEC	NORO 506	33.9669, -83.3906	mTEC	0	B2	N/A	Pan-susceptible
	25 mTEC	NORO 510	33.957, -83.3665	mTEC	450	B1	N/A	Tet
	26 mTEC	NORO 527	33.9756, -83.3927	mTEC	350	B1	N/A	Pan-susceptible
	27 mTEC	NORO 605	33.9323, -83.3594	mTEC	1100	C	N/A	StrSulTet
	28 mTEC	NORO 607	33.955, -83.3808	mTEC	3500	F	N/A	Pan-susceptible
	29 mTEC	NORO 609	33.9077, -83.3599	mTEC	1250	B1	N/A	Pan-susceptible
	30 mTEC	NORO 618	33.9481, -83.3799	mTEC	1150	B1	N/A	Pan-susceptible
	1 ECC	BICO 201	33.969265, -83.303852	ECC	350	A	N/A	Pan-susceptible
	2 ECC	MIDO 101	33.9987, -83.4954	ECC	0	B1	N/A	Pan-susceptible
	3 ECC	MIDO 103	34.0006, -83.503	ECC	150	B2	N/A	Pan-susceptible
	4 ECC	MIDO 305	33.996, -83.5639	ECC	300	B1	N/A	Pan-susceptible
	5 ECC	MIDO 601	33.926339, -83.426795	ECC	0	E	N/A	Pan-susceptible
	6 ECC	MIDO 604	33.9232, -83.441	ECC	550	B2	N/A	Pan-susceptible
	7 ECC	MIDO 605	33.931, -83.4877	ECC	50	A	N/A	Pan-susceptible
	8 ECC	MIDO 606	33.9298, -83.4798	ECC	200	B2	N/A	Pan-susceptible
	9 ECC	MIDO 608	33.9267, -83.4612	ECC	100	B2	N/A	Pan-susceptible
	10 ECC	MIDO 609	33.9301, -83.5517	ECC	450	E	N/A	Pan-susceptible
	11 ECC	MIDO 610	33.9245, -83.4549	ECC	100	B2	N/A	Pan-susceptible
	12 ECC	MIDO 611	33.9313, -83.5086	ECC	50	B1	EPEC	Pan-susceptible
	13 ECC	MIDO 612	33.9194, -83.4159	ECC	0	C	N/A	Tet
	14 ECC	MIDO 613	33.9255, -83.5226	ECC	100	E	N/A	Pan-susceptible
	17 ECC	MIDO 704	33.903, -83.3797	ECC	100	A	N/A	Pan-susceptible
	18 ECC	MIDO 708	33.900855, -83.379822	ECC	0	B2	N/A	Pan-susceptible
	19 ECC	MIDO 801	33.957765, -83.438489	ECC	200	E	N/A	Pan-susceptible
	20 ECC	MIDO 802	33.958, -83.4368	ECC	100	E	N/A	Pan-susceptible
	21 ECC	MIDO 804	33.9743, -83.425	ECC	200	B2	N/A	Pan-susceptible
	22 ECC	MIDO 805	33.9746, -83.4241	ECC	100	E	N/A	Pan-susceptible
	23 ECC	NORO 503	33.9545, -83.3659	ECC	0	B2	N/A	Pan-susceptible
	24 ECC	NORO 506	33.9669, -83.3906	ECC	0	A	N/A	Pan-susceptible
	25 ECC	NORO 510	33.957, -83.3665	ECC	450	A	N/A	Tet
	26 ECC	NORO 527	33.9756, -83.3927	ECC	350	B1	N/A	Pan-susceptible
	27 ECC	NORO 605	33.9323, -83.3594	ECC	1100	B1	N/A	Tet
	28 ECC	NORO 607	33.955, -83.3808	ECC	3500	E	N/A	Pan-susceptible
	29 ECC	NORO 609	33.9077, -83.3599	ECC	1250	C	N/A	Tet
	30 ECC	NORO 618	33.9481, -83.3799	ECC	1150	A	N/A	Pan-susceptible
2015 Spring	31 ECC	BICO 101	33.8556, -83.3266	ECC	150	F	N/A	Pan-susceptible
	32 ECC	BICO 201	33.969265, -83.303852	ECC	200	E	N/A	Pan-susceptible
	33 ECC	MIDO 101	33.9987, -83.4954	ECC	50	E	N/A	Pan-susceptible
	34 ECC	MIDO 103	34.0006, -83.503	ECC	450	B2	N/A	Pan-susceptible
	35 ECC	MIDO 301	33.967564, -83.497176	ECC	50	B2	N/A	Pan-susceptible
	36 ECC	MIDO 305	33.996, -83.5639	ECC	1050	B1	N/A	Pan-susceptible
	37 ECC	MIDO 316	33.9659, -83.515	ECC	150	B1	N/A	Pan-susceptible
	38 ECC	MIDO 318	33.9655, -83.5222	ECC	150	E	N/A	Pan-susceptible
	39 ECC	MIDO 502	33.8992, -83.4428	ECC	0	B2	N/A	Pan-susceptible
	40 ECC	MIDO 504	33.9112, -83.4057	ECC	400	B1	N/A	StrSulTet
	41 ECC	MIDO 505	33.9025, -83.5232	ECC	750	A	N/A	Pan-susceptible
	42 ECC	MIDO 506	33.8976, -83.4332	ECC	100	A	N/A	Pan-susceptible
	43 ECC	MIDO 507	33.8938, -83.5046	ECC	50	B1	N/A	Pan-susceptible
	44 ECC	MIDO 508	33.8944, -83.5028	ECC	650	B1	N/A	Pan-susceptible
	45 ECC	MIDO 509	33.8898, -83.4572	ECC	50	B2	N/A	Pan-susceptible

46 ECC	MIDO 510	33.9023, -83.4643	ECC	400	B2	N/A	Pan-susceptible
47 ECC	MIDO 511	33.9039, -83.4632	ECC	600	A	N/A	Pan-susceptible
48 ECC	MIDO 512	33.9082, -83.4142	ECC	0	B1	N/A	Pan-susceptible
49 ECC	MIDO 515	33.8965, -83.4613	ECC	100	B2	N/A	Pan-susceptible
50 ECC	MIDO 601	33.926339, -83.426795	ECC	350	B2	N/A	Pan-susceptible
51 ECC	MIDO 604	33.9232, -83.441	ECC	200	B2	N/A	Pan-susceptible
52 ECC	MIDO 605	33.931, -83.4877	ECC	400	B2	N/A	Pan-susceptible
53 ECC	MIDO 606	33.9298, -83.4798	ECC	400	B2	N/A	Pan-susceptible
54 ECC	MIDO 608	33.9267, -83.4612	ECC	500	B2	N/A	Pan-susceptible
55 ECC	MIDO 609	33.9301, -83.5517	ECC	350	B1	N/A	Pan-susceptible
56 ECC	MIDO 610	33.9245, -83.4549	ECC	500	B1	N/A	Pan-susceptible
57 ECC	MIDO 611	33.9313, -83.5086	ECC	500	E	N/A	Pan-susceptible
58 ECC	MIDO 612	33.9194, -83.4159	ECC	650	B2	N/A	Pan-susceptible
59 ECC	MIDO 613	33.9255, -83.5226	ECC	300	E	N/A	Pan-susceptible
60 ECC	MIDO 614	33.9132, -83.4034	ECC	200	E	N/A	Pan-susceptible
61 ECC	MIDO 616	33.9296, -83.4567	ECC	300	E	N/A	Pan-susceptible
62 ECC	MIDO 617	33.9339, -83.5459	ECC	400	B2	N/A	Pan-susceptible
63 ECC	MIDO 702	33.911, -83.402	ECC	200	E	N/A	Pan-susceptible
64 ECC	MIDO 704	33.903, -83.3797	ECC	0	A	N/A	Pan-susceptible
65 ECC	MIDO 706	33.8684, -83.4194	ECC	1150	C	N/A	Tet
66 ECC	MIDO 707	33.8818, -83.3896	ECC	2350	C	N/A	Nal
67 ECC	MIDO 708	33.900855, -83.379822	ECC	0	B2	N/A	Pan-susceptible
68 ECC	MIDO 709	33.8664, -83.4036	ECC	200	E	N/A	Pan-susceptible
69 ECC	MIDO 712	33.867468, -83.416044	ECC	450	B2	N/A	Pan-susceptible
70 ECC	MIDO 718	33.9002, -83.3977	ECC	0	E	N/A	Pan-susceptible
71 ECC	MIDO 719	33.8906, -83.3765	ECC	100	B2	N/A	Pan-susceptible
72 ECC	MIDO 801	33.957765, -83.438489	ECC	200	B2	N/A	Pan-susceptible
73 ECC	MIDO 802	33.958, -83.4368	ECC	300	B2	N/A	Pan-susceptible
74 ECC	MIDO 804	33.9743, -83.425	ECC	200	B2	EPEC	Pan-susceptible
75 ECC	MIDO 805	33.9746, -83.4241	ECC	100	E	N/A	Pan-susceptible
76 ECC	MIDO 806	33.952876, -83.480387	ECC	0	E	N/A	Pan-susceptible
77 ECC	MIDO 807	33.9661, -83.4312	ECC	100	B2	N/A	Pan-susceptible
78 ECC	MIDO 812	33.9347, -83.4084	ECC	700	B1	N/A	Pan-susceptible
79 ECC	MIDO 813	33.9638, -83.444	ECC	50	B2	N/A	Pan-susceptible
80 ECC	MIDO 814	33.94, -83.4091	ECC	400	B1	N/A	Pan-susceptible
81 ECC	MIDO 816	33.9744, -83.4539	ECC	250	E	N/A	Pan-susceptible
82 ECC	MIDO 817	33.988018, -83.448036	ECC	250	B1	N/A	Pan-susceptible
83 ECC	MIDO 818	33.936785, -83.402523	ECC	950	A	N/A	Pan-susceptible
84 ECC	MIDO 820	33.9893, -83.457	ECC	50	B2	N/A	Pan-susceptible
85 ECC	MIDO 821	33.956178, -83.457845	ECC	300	B2	N/A	Pan-susceptible
86 ECC	MIDO 825	33.9576, -83.4006	ECC	450	B2	N/A	Pan-susceptible
87 ECC	MIDO 826	33.9558, -83.4005	ECC	200	E	N/A	Pan-susceptible
88 ECC	MIDO 828	33.9246, -83.3896	ECC	550	F	N/A	Pan-susceptible
89 ECC	MIDO 831	33.9418, -83.4016	ECC	500	B1	N/A	Pan-susceptible
90 ECC	NORO 102	34.0337, -83.4399	ECC	250	B2	N/A	Pan-susceptible
91 ECC	NORO 108	33.994, -83.4007	ECC	100	C	N/A	Pan-susceptible
92 ECC	NORO 109	34.029, -83.4744	ECC	200	B1	N/A	Pan-susceptible
93 ECC	NORO 110	34.0672, -83.463	ECC	250	B2	N/A	Pan-susceptible
94 ECC	NORO 111	34.0211, -83.3687	ECC	150	B2	N/A	Pan-susceptible
95 ECC	NORO 114	34.0106, -83.4067	ECC	100	E	N/A	Tet
96 ECC	NORO 115	34.0293, -83.4619	ECC	250	B1	N/A	Pan-susceptible
97 ECC	NORO 117	34.048307, -83.473621	ECC	600	B2	N/A	Pan-susceptible
98 ECC	NORO 201	34.1266, -83.3944	ECC	300	B2	N/A	Pan-susceptible
99 ECC	NORO 301	34.0582, -83.389	ECC	150	B1	N/A	Pan-susceptible
100 ECC	NORO 401	33.9805, -83.382	ECC	0	E	N/A	Pan-susceptible
101 ECC	NORO 402	34.0647, -83.3659	ECC	500	B2	N/A	Pan-susceptible
102 ECC	NORO 403	34.0634, -83.3452	ECC	250	E	N/A	Pan-susceptible
104 ECC	NORO 407	34.0211, -83.3687	ECC	0	B2	N/A	Pan-susceptible
105 ECC	NORO 501	33.9772, -83.3318	ECC	400	E	N/A	Pan-susceptible
106 ECC	NORO 502	33.9692, -83.3883	ECC	500	B2	N/A	Pan-susceptible
107 ECC	NORO 503	33.9545, -83.3659	ECC	650	E	N/A	Amp
108 ECC	NORO 504	33.9526, -83.3594	ECC	100	B2	N/A	Pan-susceptible
109 ECC	NORO 505	33.9731, -83.3328	ECC	5150	B2	N/A	Pan-susceptible
110 ECC	NORO 506	33.9669, -83.3906	ECC	0	E	N/A	Pan-susceptible
111 ECC	NORO 510	33.957, -83.3665	ECC	200	E	N/A	Pan-susceptible
112 ECC	NORO 511	33.9785, -83.3379	ECC	50	E	N/A	Pan-susceptible
113 ECC	NORO 512	33.9772, -83.3278	ECC	0	B1	N/A	Pan-susceptible
114 ECC	NORO 513	33.9891, -83.3508	ECC	250	E	N/A	Pan-susceptible
115 ECC	NORO 514	33.974, -83.3524	ECC	100	B1	N/A	Pan-susceptible
116 ECC	NORO 515	33.9606, -83.3626	ECC	300	B1	N/A	Pan-susceptible
117 ECC	NORO 517	33.968, -83.3792	ECC	150	B1	N/A	Pan-susceptible
118 ECC	NORO 518	33.966445, -83.397939	ECC	7000	B2	N/A	Pan-susceptible
119 ECC	NORO 519	33.9914, -83.3141	ECC	100	B2	EPEC	Pan-susceptible

	120 ECC	NORO 520	33.9764, -83.3899	ECC	600	B2	N/A	Pan-susceptible
	121 ECC	NORO 601	33.9474, -83.3526	ECC	0	B2	N/A	Pan-susceptible
	122 ECC	NORO 603	33.9364, -83.3523	ECC	0	B1	N/A	Pan-susceptible
	123 ECC	NORO 604	33.9455, -83.3542	ECC	0	B2	N/A	Pan-susceptible
	124 ECC	NORO 605	33.9323, -83.3594	ECC	150	B2	EPEC	Pan-susceptible
	125 ECC	NORO 607	33.955, -83.3808	ECC	1850	A	N/A	Pan-susceptible
	126 ECC	NORO 609	33.9077, -83.3599	ECC	0	F	N/A	Pan-susceptible
	127 ECC	NORO 610	33.9522, -83.3687	ECC	250	E	N/A	Pan-susceptible
	128 ECC	NORO 611	33.9435, -83.3297	ECC	100	E	N/A	Pan-susceptible
	129 ECC	NORO 615	33.8904, -83.3534	ECC	150	B1	N/A	Pan-susceptible
	130 ECC	NORO 618	33.9481, -83.3799	ECC	550	A	N/A	Pan-susceptible
2015 Summer	131 ECC	BICO 101	33.8556, -83.3266	ECC	100	B1	N/A	Pan-susceptible
	132 ECC	Calls creek @ Harris Shoals	33.868465, -83.419163	ECC	50	B1	N/A	Pan-susceptible
	133 ECC	MIDO 301	33.967564, -83.497176	ECC	100	F	N/A	Pan-susceptible
	134 ECC	MIDO 504	33.9112, -83.4057	ECC	0	E	N/A	Pan-susceptible
	135 ECC	MIDO 604	33.9232, -83.441	ECC	0	E	N/A	Pan-susceptible
	136 ECC	MIDO 605	33.931, -83.4877	ECC	100	B1	N/A	Pan-susceptible
	137 ECC	MIDO 606	33.9298, -83.4798	ECC	50	B1	N/A	Pan-susceptible
	138 ECC	MIDO 608	33.9267, -83.4612	ECC	150	E	N/A	Pan-susceptible
	139 ECC	MIDO 609	33.9301, -83.5517	ECC	650	B1	N/A	Pan-susceptible
	140 ECC	MIDO 610	33.9245, -83.4549	ECC	150	B2	N/A	Pan-susceptible
	141 ECC	MIDO 611	33.9313, -83.5086	ECC	0	B1	N/A	Pan-susceptible
	142 ECC	MIDO 612	33.9194, -83.4159	ECC	0	B1	N/A	Pan-susceptible
	143 ECC	MIDO 613	33.9255, -83.5226	ECC	200	E	N/A	Pan-susceptible
	144 ECC	MIDO 614	33.9132, -83.4034	ECC	200	E	N/A	Pan-susceptible
	145 ECC	MIDO 616	33.9296, -83.4567	ECC	100	E	N/A	Pan-susceptible
	146 ECC	MIDO 617	33.9339, -83.5459	ECC	200	F	N/A	Pan-susceptible
	147 ECC	MIDO 702	33.911, -83.402	ECC	200	B1	N/A	Pan-susceptible
	148 ECC	MIDO 706	33.8684, -83.4194	ECC	0	B1	N/A	Pan-susceptible
	149 ECC	MIDO 707	33.8818, -83.3896	ECC	100	B1	N/A	Pan-susceptible
	150 ECC	MIDO 709	33.8664, -83.4036	ECC	150	E	N/A	Pan-susceptible
	151 ECC	MIDO 719	33.8906, -83.3765	ECC	0	A	N/A	Pan-susceptible
	152 ECC	MIDO 801	33.957765, -83.438489	ECC	0	B1	EPEC	Pan-susceptible
	153 ECC	MIDO 802	33.958, -83.4368	ECC	400	B1	N/A	Pan-susceptible
	154 ECC	MIDO 804	33.9743, -83.425	ECC	250	C	EPEC	Pan-susceptible
	155 ECC	MIDO 805	33.9746, -83.4241	ECC	450	B1	N/A	Pan-susceptible
	156 ECC	MIDO 812	33.9347, -83.4084	ECC	150	B1	N/A	Pan-susceptible
	157 ECC	MIDO 818	33.936785, -83.402523	ECC	200	E	N/A	Pan-susceptible
	158 ECC	MIDO 825	33.9576, -83.4006	ECC	250	E	N/A	Pan-susceptible
	159 ECC	MIDO 826	33.9558, -83.4005	ECC	400	A	EPEC	Pan-susceptible
	160 ECC	MIDO 828	33.9246, -83.3896	ECC	2000	B1	N/A	Pan-susceptible
	161 ECC	NORO 503	33.9545, -83.3659	ECC	1800	B2	N/A	Amp
	162 ECC	NORO 510	33.957, -83.3665	ECC	150	E	N/A	Pan-susceptible
	139a mTEC	MIDO 609	33.9301, -83.5517	mTEC	650	B1	N/A	Pan-susceptible
	139b mTEC	MIDO 609	33.9301, -83.5517	mTEC	650	B1	N/A	Pan-susceptible
	143a mTEC	MIDO 613	33.9255, -83.5226	mTEC	200	B2	N/A	Pan-susceptible
	143c mTEC	MIDO 613	33.9255, -83.5226	mTEC	200	E	N/A	Pan-susceptible
	144b mTEC	MIDO 614	33.9132, -83.4034	mTEC	200	E	N/A	Pan-susceptible
	144c mTEC	MIDO 614	33.9132, -83.4034	mTEC	200	B2	N/A	Pan-susceptible
	145a mTEC	MIDO 616	33.9296, -83.4567	mTEC	100	E	N/A	Pan-susceptible
	145c mTEC	MIDO 616	33.9296, -83.4567	mTEC	100	B1	N/A	Pan-susceptible
	149a mTEC	MIDO 707	33.8818, -83.3896	mTEC	100	B2	N/A	Pan-susceptible
	149c mTEC	MIDO 707	33.8818, -83.3896	mTEC	100	E	N/A	Pan-susceptible
	153a mTEC	MIDO 802	33.958, -83.4368	mTEC	400	B1	N/A	Pan-susceptible
	155a mTEC	MIDO 805	33.9746, -83.4241	mTEC	450	B1	N/A	Pan-susceptible
	159a mTEC	MIDO 826	33.9558, -83.4005	mTEC	400	B1	N/A	Pan-susceptible
	159c mTEC	MIDO 826	33.9558, -83.4005	mTEC	400	E	N/A	Tet
2015 Fall	164 ECC	BICO 101	33.8556, -83.3266	ECC	200	B2	N/A	AmpNal
	165 ECC	BICO 201	33.969265, -83.303852	ECC	200	F	N/A	Pan-susceptible
	166 ECC	MIDO 101	33.9987, -83.4954	ECC	0	E	N/A	Pan-susceptible
	167 ECC	MIDO 103	34.0006, -83.503	ECC	0	B2	N/A	Pan-susceptible
	168 ECC	MIDO 301	33.967564, -83.497176	ECC	0	E	N/A	Pan-susceptible
	169 ECC	MIDO 305	33.996, -83.5639	ECC	200	B1	N/A	Pan-susceptible
	170 ECC	MIDO 504	33.9112, -83.4057	ECC	100	B2	N/A	Pan-susceptible
	171 ECC	MIDO 505	33.9025, -83.5232	ECC	50	B1	N/A	Tet
	172 ECC	MIDO 507	33.8938, -83.5046	ECC	0	E	N/A	Pan-susceptible
	173 ECC	MIDO 508	33.8944, -83.5028	ECC	100	F	N/A	Pan-susceptible
	174 ECC	MIDO 509	33.8898, -83.4572	ECC	0	B2	N/A	Pan-susceptible
	175 ECC	MIDO 510	33.9023, -83.4643	ECC	50	B2	N/A	Pan-susceptible
	176 ECC	MIDO 511	33.9039, -83.4632	ECC	0	B1	N/A	Pan-susceptible
	177 ECC	MIDO 515	33.8965, -83.4613	ECC	0	B1	N/A	Pan-susceptible
	178 ECC	MIDO 601	33.926339, -83.426795	ECC	0	B1	N/A	Pan-susceptible
	179 ECC	MIDO 604	33.9232, -83.441	ECC	50	B2	N/A	Pan-susceptible

	180 ECC	MIDO 605	33.931, -83.4877	ECC	50	A	N/A	Pan-susceptible
	181 ECC	MIDO 606	33.9298, -83.4798	ECC	200	E	N/A	Pan-susceptible
	182 ECC	MIDO 608	33.9267, -83.4612	ECC	50	B2	N/A	Pan-susceptible
	183 ECC	MIDO 609	33.9301, -83.5517	ECC	100	B1	N/A	Pan-susceptible
	184 ECC	MIDO 610	33.9245, -83.4549	ECC	50	B2	N/A	Pan-susceptible
	185 ECC	MIDO 611	33.9313, -83.5086	ECC	100	A	N/A	Pan-susceptible
	186 ECC	MIDO 612	33.9194, -83.4159	ECC	50	E	N/A	Pan-susceptible
	187 ECC	MIDO 613	33.9255, -83.5226	ECC	150	B1	N/A	Pan-susceptible
	188 ECC	MIDO 616	33.9296, -83.4567	ECC	150	B1	N/A	Pan-susceptible
	189 ECC	MIDO 617	33.9339, -83.5459	ECC	50	A	N/A	Pan-susceptible
	190 ECC	MIDO 704	33.903, -83.3797	ECC	0	B1	N/A	Pan-susceptible
	191 ECC	MIDO 706	33.8684, -83.4194	ECC	50	E	N/A	Pan-susceptible
	192 ECC	MIDO 707	33.8818, -83.3896	ECC	150	B2	N/A	ChlStrSulTet
	193 ECC	MIDO 708	33.900855, -83.379822	ECC	0	B1	STEC	Pan-susceptible
	194 ECC	MIDO 709	33.8664, -83.4036	ECC	150	B2	EPEC	Pan-susceptible
	195 ECC	MIDO 712	33.867468, -83.416044	ECC	50	B2	EPEC	Pan-susceptible
	196 ECC	MIDO 719	33.8906, -83.3765	ECC	200	B1	N/A	Pan-susceptible
	197 ECC	MIDO 802	33.958, -83.4368	ECC	100	B2	N/A	Pan-susceptible
	198 ECC	MIDO 803	33.9476, -83.3995	ECC	0	E	N/A	Pan-susceptible
	199 ECC	MIDO 804	33.9743, -83.425	ECC	50	B1	N/A	Pan-susceptible
	200 ECC	MIDO 805	33.9746, -83.4241	ECC	50	B1	N/A	Pan-susceptible
	201 ECC	MIDO 807	33.9661, -83.4312	ECC	100	B2	EPEC	Pan-susceptible
	202 ECC	MIDO 811	33.9567, -83.4177	ECC	50	B2	EPEC	Pan-susceptible
	203 ECC	MIDO 812	33.9347, -83.4084	ECC	150	A	N/A	Pan-susceptible
	204 ECC	MIDO 813	33.9638, -83.444	ECC	0	B1	N/A	Pan-susceptible
	205 ECC	MIDO 814	33.94, -83.4091	ECC	0	B2	N/A	Tet
	206 ECC	MIDO 818	33.936785, -83.402523	ECC	350	B2	N/A	Pan-susceptible
	207 ECC	MIDO 821	33.956178, -83.457845	ECC	0	E	N/A	Amp
	208 ECC	MIDO 825	33.9576, -83.4006	ECC	150	E	N/A	Pan-susceptible
	209 ECC	MIDO 826	33.9558, -83.4005	ECC	150	B1	N/A	Pan-susceptible
	210 ECC	MIDO 828	33.9246, -83.3896	ECC	150	B2	N/A	Pan-susceptible
	211 ECC	MIDO 831	33.9418, -83.4016	ECC	250	B1	N/A	Pan-susceptible
	212 ECC	NORO 502	33.9692, -83.3883	ECC	200	B1	N/A	Pan-susceptible
	213 ECC	NORO 503	33.9545, -83.3659	ECC	1650	B1	N/A	Pan-susceptible
	214 ECC	NORO 517	33.968, -83.3792	ECC	400	B1	N/A	Pan-susceptible
	215 ECC	NORO 518	33.966445, -83.397939	ECC	100	B1	N/A	Pan-susceptible
	216 ECC	NORO 601	33.9474, -83.3526	ECC	0	E	N/A	Pan-susceptible
	217 ECC	NORO 603	33.9364, -83.3523	ECC	0	F	N/A	Pan-susceptible
	218 ECC	NORO 604	33.9455, -83.3542	ECC	0	B2	N/A	Pan-susceptible
	219 ECC	NORO 605	33.9323, -83.3594	ECC	100	B2	EPEC	Pan-susceptible
	220 ECC	NORO 609	33.9077, -83.3599	ECC	150	B1	N/A	Pan-susceptible
	221 ECC	NORO 611	33.9435, -83.3297	ECC	100	B2	N/A	Pan-susceptible
	222 ECC	NORO 627	33.9297, -83.3432	ECC	0	E	N/A	Pan-susceptible
2016 Winter	223 ECC	BICO 101	33.8556, -83.3266	ECC	550	B1	N/A	Pan-susceptible
	224 ECC	MIDO 609	33.9301, -83.5517	ECC	200	B1	N/A	Pan-susceptible
	225 ECC	MIDO 611	33.9313, -83.5086	ECC	0	B1	N/A	Pan-susceptible
	226 ECC	MIDO 605	33.931, -83.4877	ECC	50	B1	N/A	Pan-susceptible
	227 ECC	MIDO 606	33.9298, -83.4798	ECC	100	B1	N/A	Pan-susceptible
	228 ECC	MIDO 608	33.9267, -83.4612	ECC	0	E	N/A	Pan-susceptible
	229 ECC	MIDO 617	33.9339, -83.5459	ECC	50	B1	N/A	Pan-susceptible
	230 ECC	MIDO 613	33.9255, -83.5226	ECC	100	B1	N/A	Pan-susceptible
	231 ECC	MIDO 708	33.900855, -83.379822	ECC	0	B1	N/A	Pan-susceptible
	232 ECC	MIDO 802	33.958, -83.4368	ECC	100	B1	N/A	Pan-susceptible
	233 ECC	MIDO 704	33.903, -83.3797	ECC	0	E	N/A	Pan-susceptible
	234 ECC	MIDO 801	33.957765, -83.438489	ECC	200	B2	N/A	Pan-susceptible
	235 ECC	MIDO 719	33.8906, -83.3765	ECC	300	B1	N/A	Pan-susceptible
	236 ECC	MIDO 612	33.9194, -83.4159	ECC	50	A	N/A	Pan-susceptible
	237 ECC	MIDO 707	33.8818, -83.3896	ECC	100	B2	N/A	Pan-susceptible
	238 ECC	MIDO 604	33.9232, -83.441	ECC	100	E	N/A	AmpAziStrSulTetTri
	239 ECC	MIDO 614	33.9132, -83.4034	ECC	350	E	N/A	Pan-susceptible
	240 ECC	MIDO 702	33.911, -83.402	ECC	0	U	N/A	Pan-susceptible
	241 ECC	MIDO 811	33.9567, -83.4177	ECC	50	B1	N/A	Pan-susceptible
	242 ECC	MIDO 825	33.9576, -83.4006	ECC	150	B2	N/A	Pan-susceptible
	243 ECC	MIDO 826	33.9558, -83.4005	ECC	100	B2	N/A	Pan-susceptible
	244 ECC	MIDO 712	33.867468, -83.416044	ECC	0	B2	N/A	Pan-susceptible
	245 ECC	MIDO 706	33.8684, -83.4194	ECC	0	B2	N/A	Pan-susceptible
	246 ECC	MIDO 709	33.8664, -83.4036	ECC	0	B2	N/A	Pan-susceptible
	247 ECC	NORO 520	33.9764, -83.3899	ECC	0	A	N/A	Pan-susceptible
	248 ECC	NORO 527	33.9756, -83.3927	ECC	0	E	N/A	Pan-susceptible
	249 ECC	NORO 114	34.0106, -83.4067	ECC	300	E	N/A	Pan-susceptible
	250 ECC	NORO 108	33.994, -83.4007	ECC	0	C	N/A	Pan-susceptible
	251 ECC	NORO 502	33.9692, -83.3883	ECC	100	B2	EPEC	Pan-susceptible
	252 ECC	NORO 517	33.968, -83.3792	ECC	0	B1	N/A	Pan-susceptible

	253 ECC	NORO 518	33.966445, -83.397939	ECC	100	B2	EPEC	Pan-susceptible
	254 ECC	NORO 510	33.957, -83.3665	ECC	150	B1	N/A	Pan-susceptible
	255 ECC	NORO 503	33.9545, -83.3659	ECC	1900	B2	N/A	Tet
	256 ECC	NORO 401	33.9805, -83.382	ECC	850	B1	N/A	Pan-susceptible
	257 ECC	NORO 609	33.9077, -83.3599	ECC	550	B1	N/A	Pan-susceptible
	258 ECC	NORO 603	33.9364, -83.3523	ECC	0	F	N/A	Pan-susceptible
	259 ECC	NORO 604	33.9455, -83.3542	ECC	0	B1	N/A	Pan-susceptible
	260 ECC	NORO 615	33.8904, -83.3534	ECC	300	B2	N/A	Pan-susceptible
	261 ECC	NORO 627	33.9297, -83.3432	ECC	0	B1	N/A	Pan-susceptible
	262 ECC	NORO 611	33.9435, -83.3297	ECC	0	B1	N/A	Pan-susceptible
	263 ECC	NORO 605	33.9323, -83.3594	ECC	750	B1	N/A	Pan-susceptible
2016 Spring	264 ECC	BICO 101	33.8556, -83.3266	ECC	750	A	N/A	SulTet
	265 ECC	BICO 201	33.969265, -83.303852	ECC	350	B1	N/A	Pan-susceptible
	266 ECC	MIDO 101	33.9987, -83.4954	ECC	50	B2	N/A	Pan-susceptible
	267 ECC	MIDO 301	33.967564, -83.497176	ECC	100	B1	N/A	Pan-susceptible
	268 ECC	MIDO 305	33.996, -83.5639	ECC	2350	B1	N/A	Pan-susceptible
	269 ECC	MIDO 502	33.8992, -83.4428	ECC	100	B1	N/A	Pan-susceptible
	270 ECC	MIDO 503	33.9097, -83.4141	ECC	100	B2	N/A	Pan-susceptible
	271 ECC	MIDO 504	33.9112, -83.4057	ECC	250	B1	N/A	Pan-susceptible
	272 ECC	MIDO 505	33.9025, -83.5232	ECC	100	B2	N/A	Pan-susceptible
	273 ECC	MIDO 506	33.8976, -83.4332	ECC	0	B2	N/A	Pan-susceptible
	274 ECC	MIDO 507	33.8938, -83.5046	ECC	550	A	N/A	AmpStrTet
	275 ECC	MIDO 508	33.8944, -83.5028	ECC	0	B2	N/A	Pan-susceptible
	276 ECC	MIDO 509	33.8898, -83.4572	ECC	150	B2	N/A	Pan-susceptible
	277 ECC	MIDO 511	33.9039, -83.4632	ECC	400	B1	N/A	Pan-susceptible
	278 ECC	MIDO 512	33.9082, -83.4142	ECC	450	B2	N/A	Pan-susceptible
	279 ECC	MIDO 515	33.8965, -83.4613	ECC	250	B1	N/A	StrSulTet
	280 ECC	MIDO 601	33.926339, -83.426795	ECC	12000	B2	N/A	AmpNal
	281 ECC	MIDO 604	33.9232, -83.441	ECC	200	B1	N/A	Tet
	282 ECC	MIDO 605	33.931, -83.4877	ECC	1200	B2	N/A	Pan-susceptible
	283 ECC	MIDO 606	33.9298, -83.4798	ECC	600	E	N/A	Pan-susceptible
	284 ECC	MIDO 608	33.9267, -83.4612	ECC	100	B2	N/A	Pan-susceptible
	285 ECC	MIDO 609	33.9301, -83.5517	ECC	1050	B1	N/A	Pan-susceptible
	286 ECC	MIDO 610	33.9245, -83.4549	ECC	1050	E	N/A	Pan-susceptible
	287 ECC	MIDO 611	33.9313, -83.5086	ECC	550	B2	N/A	Pan-susceptible
	288 ECC	MIDO 612	33.9194, -83.4159	ECC	5000	E	N/A	Pan-susceptible
	289 ECC	MIDO 613	33.9255, -83.5226	ECC	1000	B2	N/A	Pan-susceptible
	290 ECC	MIDO 614	33.9132, -83.4034	ECC	350	E	N/A	Pan-susceptible
	291 ECC	MIDO 616	33.9296, -83.4567	ECC	350	B2	N/A	Pan-susceptible
	292 ECC	MIDO 617	33.9339, -83.5459	ECC	100	E	N/A	Pan-susceptible
	293 ECC	MIDO 702	33.911, -83.402	ECC	1100	B1	N/A	Pan-susceptible
	294 ECC	MIDO 704	33.903, -83.3797	ECC	50	E	N/A	Pan-susceptible
	295 ECC	MIDO 706	33.8684, -83.4194	ECC	100	B1	N/A	Pan-susceptible
	296 ECC	MIDO 707	33.8818, -83.3896	ECC	450	F	N/A	Pan-susceptible
	297 ECC	MIDO 709	33.8664, -83.4036	ECC	50	B2	N/A	Pan-susceptible
	298 ECC	MIDO 712	33.867468, -83.416044	ECC	300	E	N/A	Pan-susceptible
	299 ECC	MIDO 718	33.9002, -83.3977	ECC	50	B2	N/A	Pan-susceptible
	300 ECC	MIDO 719	33.8906, -83.3765	ECC	1400	E	N/A	Pan-susceptible
	301 ECC	MIDO 802	33.958, -83.4368	ECC	300	E	N/A	Pan-susceptible
	302 ECC	MIDO 804	33.9743, -83.425	ECC	650	B1	N/A	Pan-susceptible
	303 ECC	MIDO 805	33.9746, -83.4241	ECC	200	B2	N/A	AmoAmpFoxTioAxoGen
	304 ECC	MIDO 806	33.952876, -83.480387	ECC	250	E	N/A	Pan-susceptible
	305 ECC	MIDO 811	33.9567, -83.4177	ECC	200	B2	N/A	Pan-susceptible
	306 ECC	MIDO 813	33.9638, -83.444	ECC	700	C	N/A	Pan-susceptible
	307 ECC	MIDO 814	33.94, -83.4091	ECC	0	B1	N/A	Pan-susceptible
	308 ECC	MIDO 816	33.9744, -83.4539	ECC	300	B1	N/A	Pan-susceptible
	309 ECC	MIDO 817	33.988018, -83.448036	ECC	0	B2	N/A	Pan-susceptible
	310 ECC	MIDO 818	33.936785, -83.402523	ECC	50	B2	N/A	Pan-susceptible
	311 ECC	MIDO 820	33.9893, -83.457	ECC	200	A	N/A	Pan-susceptible
	312 ECC	MIDO 821	33.956178, -83.457845	ECC	450	F	N/A	Pan-susceptible
	313 ECC	MIDO 825	33.9576, -83.4006	ECC	750	B2	N/A	Pan-susceptible
	314 ECC	MIDO 826	33.9558, -83.4005	ECC	700	B2	N/A	Pan-susceptible
	315 ECC	MIDO 828	33.9246, -83.3896	ECC	150	A	N/A	Pan-susceptible
	316 ECC	MIDO 831	33.9418, -83.4016	ECC	650	B2	N/A	Pan-susceptible
	317 ECC	NORO 108	33.994, -83.4007	ECC	400	B1	N/A	Pan-susceptible
	318 ECC	NORO 109	34.029, -83.4744	ECC	150	B2	N/A	Pan-susceptible
	319 ECC	NORO 110	34.0672, -83.463	ECC	2200	B2	N/A	Pan-susceptible
	320 ECC	NORO 111	34.0211, -83.3687	ECC	150	B2	N/A	Pan-susceptible
	321 ECC	NORO 114	34.0106, -83.4067	ECC	700	B1	N/A	Pan-susceptible
	322 ECC	NORO 115	34.0293, -83.4619	ECC	0	B2	N/A	Pan-susceptible
	323 ECC	NORO 117	34.048307, -83.473621	ECC	50	B2	N/A	Pan-susceptible
	324 ECC	NORO 201	34.1266, -83.3944	ECC	50	F	N/A	Pan-susceptible
	325 ECC	NORO 301	34.0582, -83.389	ECC	200	A	N/A	Pan-susceptible

	326 ECC	NORO 401	33.9805, -83.382	ECC	100	F	N/A	Pan-susceptible
	327 ECC	NORO 402	34.0647, -83.3659	ECC	650	B1	N/A	Pan-susceptible
	328 ECC	NORO 403	34.0634, -83.3452	ECC	400	B2	N/A	Pan-susceptible
	329 ECC	NORO 405	34.007666, -83.364893	ECC	950	E	N/A	Pan-susceptible
	330 ECC	NORO 407	34.0211, -83.3687	ECC	150	B2	N/A	Pan-susceptible
	331 ECC	NORO 502	33.9692, -83.3883	ECC	850	B2	N/A	Pan-susceptible
	332 ECC	NORO 503	33.9545, -83.3659	ECC	2000	A	N/A	Pan-susceptible
	333 ECC	NORO 504	33.9526, -83.3594	ECC	150	B2	N/A	Pan-susceptible
	334 ECC	NORO 510	33.957, -83.3665	ECC	3100	E	N/A	Pan-susceptible
	335 ECC	NORO 515	33.9606, -83.3626	ECC	750	B1	N/A	Pan-susceptible
	336 ECC	NORO 517	33.968, -83.3792	ECC	250	E	N/A	Pan-susceptible
	337 ECC	NORO 518	33.966445, -83.397939	ECC	600	B2	N/A	Pan-susceptible
	338 ECC	NORO 520	33.9764, -83.3899	ECC	600	B2	N/A	Pan-susceptible
	339 ECC	NORO 527	33.9756, -83.3927	ECC	600	B1	N/A	AmoTet
	340 ECC	NORO 601	33.9474, -83.3526	ECC	0	B2	N/A	Pan-susceptible
	341 ECC	NORO 603	33.9364, -83.3523	ECC	100	B2	N/A	Pan-susceptible
	342 ECC	NORO 604	33.9455, -83.3542	ECC	50	B2	N/A	Pan-susceptible
	343 ECC	NORO 605	33.9323, -83.3594	ECC	300	B1	N/A	Pan-susceptible
	344 ECC	NORO 607	33.955, -83.3808	ECC	550	B2	N/A	Pan-susceptible
	345 ECC	NORO 609	33.9077, -83.3599	ECC	700	E	N/A	Pan-susceptible
	346 ECC	NORO 610	33.9522, -83.3687	ECC	2850	C	N/A	Pan-susceptible
	347 ECC	NORO 611	33.9435, -83.3297	ECC	200	B1	N/A	Pan-susceptible
	348 ECC	NORO 615	33.8904, -83.3534	ECC	550	A	N/A	Pan-susceptible
	349 ECC	NORO 618	33.9481, -83.3799	ECC	300	B2	N/A	Pan-susceptible
	350 ECC	NORO 627	33.9297, -83.3432	ECC	150	B2	N/A	Pan-susceptible
2016 Summer	351 ECC	MIDO 101	33.9987, -83.4954	ECC	300	F	N/A	Pan-susceptible
	352 ECC	MIDO 103	34.0006, -83.503	ECC	100	E	N/A	Pan-susceptible
	353 ECC	MIDO 305	33.996, -83.5639	ECC	150	B1	N/A	StrTet
	354 ECC	MIDO 605	33.931, -83.4877	ECC	150	B1	N/A	Pan-susceptible
	355 ECC	MIDO 606	33.9298, -83.4798	ECC	150	E	N/A	Pan-susceptible
	356 ECC	MIDO 608	33.9267, -83.4612	ECC	150	B1	N/A	Pan-susceptible
	357 ECC	MIDO 609	33.9301, -83.5517	ECC	450	B1	N/A	Pan-susceptible
	358 ECC	MIDO 610	33.9245, -83.4549	ECC	150	B2	EPEC	Pan-susceptible
	359 ECC	MIDO 611	33.9313, -83.5086	ECC	50	E	N/A	Pan-susceptible
	360 ECC	MIDO 612	33.9194, -83.4159	ECC	250	B1	N/A	Pan-susceptible
	361 ECC	MIDO 613	33.9255, -83.5226	ECC	600	F	N/A	Pan-susceptible
	362 ECC	MIDO 616	33.9296, -83.4567	ECC	250	B1	N/A	Pan-susceptible
	363 ECC	MIDO 617	33.9339, -83.5459	ECC	0	B1	N/A	Pan-susceptible
	364 ECC	MIDO 801	33.957765, -83.438489	ECC	250	E	N/A	Pan-susceptible
	365 ECC	MIDO 802	33.958, -83.4368	ECC	350	B2	N/A	Pan-susceptible
	366 ECC	MIDO 825	33.9576, -83.4006	ECC	7250	B2	N/A	Pan-susceptible
	367 ECC	MIDO 826	33.9558, -83.4005	ECC	1900	B2	N/A	AmpTioAxoNal
	368 ECC	NORO 108	33.994, -83.4007	ECC	450	B2	N/A	Pan-susceptible
	369 ECC	NORO 114	34.0106, -83.4067	ECC	350	U	N/A	Pan-susceptible
	370 ECC	NORO 401	33.9805, -83.382	ECC	100	B2	N/A	Pan-susceptible
	371 ECC	NORO 501	33.9772, -83.3318	ECC	450	B1	N/A	Pan-susceptible
	372 ECC	NORO 503	33.9545, -83.3659	ECC	4600	A	N/A	Pan-susceptible
	373 ECC	NORO 510	33.957, -83.3665	ECC	450	E	N/A	Pan-susceptible
	374 ECC	NORO 511	33.9785, -83.3379	ECC	350	B2	N/A	Pan-susceptible
	375 ECC	NORO 514	33.974, -83.3524	ECC	450	E	N/A	Pan-susceptible
	376 ECC	NORO 520	33.9764, -83.3899	ECC	250	E	N/A	Pan-susceptible
	377 ECC	NORO 527	33.9756, -83.3927	ECC	250	B1	N/A	Pan-susceptible
2016 Fall	378 ECC	BICO 101	33.8556, -83.3266	ECC	200	B2	N/A	Pan-susceptible
	379 ECC	BICO 201	33.969265, -83.303852	ECC	0	E	N/A	Pan-susceptible
	380 ECC	MIDO 101	33.9987, -83.4954	ECC	350	B1	N/A	Pan-susceptible
	381 ECC	MIDO 103	34.0006, -83.503	ECC	100	C	N/A	AziSulTetTri
	382 ECC	MIDO 301	33.967564, -83.497176	ECC	50	F	N/A	Tet
	383 ECC	MIDO 305	33.996, -83.5639	ECC	250	B1	N/A	Pan-susceptible
	384 ECC	MIDO 505	33.9025, -83.5232	ECC	200	E	N/A	Pan-susceptible
	385 ECC	MIDO 507	33.8938, -83.5046	ECC	200	A	N/A	Pan-susceptible
	386 ECC	MIDO 508	33.8944, -83.5028	ECC	50	E	N/A	Pan-susceptible
	387 ECC	MIDO 509	33.8898, -83.4572	ECC	2550	B1	N/A	Pan-susceptible
	388 ECC	MIDO 511	33.9039, -83.4632	ECC	50	B1	N/A	Pan-susceptible
	389 ECC	MIDO 515	33.8965, -83.4613	ECC	100	E	N/A	Pan-susceptible
	390 ECC	MIDO 601	33.926339, -83.426795	ECC	50	B1	N/A	Pan-susceptible
	391 ECC	MIDO 604	33.9232, -83.441	ECC	0	U	N/A	Pan-susceptible
	392 ECC	MIDO 605	33.931, -83.4877	ECC	200	B2	N/A	Pan-susceptible
	393 ECC	MIDO 606	33.9298, -83.4798	ECC	100	F	N/A	Pan-susceptible
	394 ECC	MIDO 608	33.9267, -83.4612	ECC	50	B2	EPEC	Pan-susceptible
	395 ECC	MIDO 609	33.9301, -83.5517	ECC	200	B1	N/A	Pan-susceptible
	396 ECC	MIDO 610	33.9245, -83.4549	ECC	250	B1	N/A	Pan-susceptible
	397 ECC	MIDO 611	33.9313, -83.5086	ECC	150	B1	N/A	Pan-susceptible
	398 ECC	MIDO 612	33.9194, -83.4159	ECC	250	E	N/A	Pan-susceptible

399 ECC	MIDO 614	33.9132, -83.4034	ECC	150	B2	N/A	Pan-susceptible
400 ECC	MIDO 616	33.9296, -83.4567	ECC	450	E	N/A	Pan-susceptible
401 ECC	MIDO 617	33.9339, -83.5459	ECC	150	B2	EPEC	Pan-susceptible
402 ECC	MIDO 702	33.911, -83.402	ECC	150	B2	N/A	Pan-susceptible
403 ECC	MIDO 704	33.903, -83.3797	ECC	0	E	N/A	Pan-susceptible
404 ECC	MIDO 706	33.8684, -83.4194	ECC	0	F	N/A	Pan-susceptible
405 ECC	MIDO 707	33.8818, -83.3896	ECC	350	F	N/A	Pan-susceptible
406 ECC	MIDO 708	33.900855, -83.379822	ECC	400	B2	N/A	Pan-susceptible
407 ECC	MIDO 709	33.8664, -83.4036	ECC	150	B1	N/A	Pan-susceptible
408 ECC	MIDO 712	33.867468, -83.416044	ECC	100	B2	N/A	Pan-susceptible
409 ECC	MIDO 719	33.8906, -83.3765	ECC	100	B2	N/A	Pan-susceptible
410 ECC	MIDO 801	33.957765, -83.438489	ECC	50	B1	N/A	Pan-susceptible
411 ECC	MIDO 802	33.958, -83.4368	ECC	300	B2	EPEC	Pan-susceptible
412 ECC	MIDO 804	33.9743, -83.425	ECC	450	E	N/A	Pan-susceptible
413 ECC	MIDO 805	33.9746, -83.4241	ECC	500	E	N/A	Pan-susceptible
414 ECC	MIDO 811	33.9567, -83.4177	ECC	450	E	N/A	Pan-susceptible
415 ECC	MIDO 816	33.9744, -83.4539	ECC	400	E	N/A	Pan-susceptible
416 ECC	MIDO 817	33.988018, -83.448036	ECC	50	E	N/A	Pan-susceptible
417 ECC	MIDO 818	33.936785, -83.402523	ECC	50	B2	N/A	Pan-susceptible
418 ECC	MIDO 820	33.9893, -83.457	ECC	0	E	N/A	Pan-susceptible
419 ECC	MIDO 825	33.9576, -83.4006	ECC	200	E	N/A	Pan-susceptible
420 ECC	MIDO 826	33.9558, -83.4005	ECC	350	B2	N/A	Pan-susceptible
421 ECC	MIDO 828	33.9246, -83.3896	ECC	50	B1	N/A	Pan-susceptible
422 ECC	MIDO 831	33.9418, -83.4016	ECC	350	B1	N/A	Pan-susceptible
423 ECC	NORO 108	33.994, -83.4007	ECC	150	A	N/A	Pan-susceptible
424 ECC	NORO 110	34.0672, -83.463	ECC	800	B1	N/A	Pan-susceptible
425 ECC	NORO 111	34.0211, -83.3687	ECC	50	F	N/A	Pan-susceptible
426 ECC	NORO 114	34.0106, -83.4067	ECC	300	B2	N/A	Pan-susceptible
427 ECC	NORO 117	34.048307, -83.473621	ECC	0	E	N/A	Pan-susceptible
428 ECC	NORO 201	34.1266, -83.3944	ECC	100	E	N/A	Pan-susceptible
429 ECC	NORO 301	34.0582, -83.389	ECC	300	E	N/A	Pan-susceptible
430 ECC	NORO 401	33.9805, -83.382	ECC	100	B1	N/A	Pan-susceptible
431 ECC	NORO 405	34.007666, -83.364893	ECC	100	B1	N/A	Pan-susceptible
432 ECC	NORO 407	34.0211, -83.3687	ECC	50	E	N/A	Pan-susceptible
433 ECC	NORO 501	33.9772, -83.3318	ECC	50	F	N/A	Pan-susceptible
434 ECC	NORO 502	33.9692, -83.3883	ECC	700	B1	N/A	Pan-susceptible
435 ECC	NORO 503	33.9545, -83.3659	ECC	50	B1	N/A	Pan-susceptible
436 ECC	NORO 504	33.9526, -83.3594	ECC	1450	A	N/A	Pan-susceptible
437 ECC	NORO 505	33.9731, -83.3328	ECC	350	B1	N/A	Pan-susceptible
438 ECC	NORO 506	33.9669, -83.3906	ECC	3850	E	N/A	Pan-susceptible
439 ECC	NORO 510	33.957, -83.3665	ECC	0	B1	N/A	Pan-susceptible
440 ECC	NORO 511	33.9785, -83.3379	ECC	150	E	N/A	Pan-susceptible
441 ECC	NORO 512	33.9772, -83.3278	ECC	300	E	N/A	Pan-susceptible
442 ECC	NORO 513	33.9891, -83.3508	ECC	100	B1	N/A	Pan-susceptible
443 ECC	NORO 514	33.974, -83.3524	ECC	350	B2	N/A	Pan-susceptible
444 ECC	NORO 515	33.9606, -83.3626	ECC	150	B2	N/A	Pan-susceptible
445 ECC	NORO 516	33.9629, -83.3805	ECC	2100	E	N/A	Pan-susceptible
446 ECC	NORO 517	33.968, -83.3792	ECC	50	E	N/A	Pan-susceptible
447 ECC	NORO 519	33.9914, -83.3141	ECC	50	A	N/A	Pan-susceptible
448 ECC	NORO 520	33.9764, -83.3899	ECC	300	B2	N/A	Pan-susceptible
449 ECC	NORO 527	33.9756, -83.3927	ECC	250	B2	N/A	Pan-susceptible
450 ECC	NORO 601	33.9474, -83.3526	ECC	50	B1	N/A	Pan-susceptible
451 ECC	NORO 603	33.9364, -83.3523	ECC	0	B2	N/A	Pan-susceptible
452 ECC	NORO 604	33.9455, -83.3542	ECC	0	B1	N/A	Pan-susceptible
453 ECC	NORO 605	33.9323, -83.3594	ECC	0	B1	N/A	Pan-susceptible
454 ECC	NORO 609	33.9077, -83.3599	ECC	0	B1	N/A	Pan-susceptible
455 ECC	NORO 610	33.9522, -83.3687	ECC	0	B1	N/A	Pan-susceptible
456 ECC	NORO 611	33.9435, -83.3297	ECC	0	E	N/A	Pan-susceptible
457 ECC	NORO 615	33.8904, -83.3534	ECC	200	E	N/A	Pan-susceptible
458 ECC	NORO 627	33.9297, -83.3432	ECC	50	E	N/A	Pan-susceptible
